# A Direct Approach to In-Plane Stress Separation using Photoelastic Ptychography

**DOI:** 10.1038/srep30541

**Published:** 2016-08-04

**Authors:** Nicholas Anthony, Guido Cadenazzi, Henry Kirkwood, Eric Huwald, Keith Nugent, Brian Abbey

**Affiliations:** 1 ARC Centre of Excellence in Advanced Molecular Imaging, Department of Chemistry and Physics, La Trobe Institute for Molecular Science, La Trobe University, Melbourne, Victoria 3086, Australia; 2 Department of Chemistry and Physics, La Trobe Institute for Molecular Science, La Trobe University, Melbourne, Victoria 3086, Australia

## Abstract

The elastic properties of materials, either under external load or in a relaxed state, influence their mechanical behaviour. Conventional optical approaches based on techniques such as photoelasticity or thermoelasticity can be used for full-field analysis of the stress distribution within a specimen. The circular polariscope in combination with holographic photoelasticity allows the sum and difference of principal stress components to be determined by exploiting the temporary birefringent properties of materials under load. Phase stepping and interferometric techniques have been proposed as a method for separating the in-plane stress components in two-dimensional photoelasticity experiments. In this paper we describe and demonstrate an alternative approach based on photoelastic ptychography which is able to obtain quantitative stress information from far fewer measurements than is required for interferometric based approaches. The complex light intensity equations based on Jones calculus for this setup are derived. We then apply this approach to the problem of a disc under diametrical compression. The experimental results are validated against the analytical solution derived by Hertz for the theoretical displacement fields for an elastic disc subject to point loading.

Determining stresses in solids is crucial for evaluating and predicting mechanical behaviour. Experimentally measuring the individual spatially varying components of the stress tensor however is often extremely challenging. The most common method of stress determination is currently Finite Element Modelling (FEM) where the solution is highly dependent on the loading and boundary conditions, both of which can be difficult to determine experimentally. The drive for new methods to measure stress in optically transparent materials, e.g. thin films, has led to the development of a variety of approaches including photoelasticity and thermoelasticity. Such full-field techniques are routinely used to obtain images of fringe patterns that *qualitatively* relate to the samples’ stress distribution. However, the data obtained using these methods typically provide either the difference or sum of the principal stress components obtained from the isochromatics (polariscope) or isopachics (holographic photoelasticity) respectively. Since the isochromatics and isopachics are directly coupled to one another, separation of the individual stress components is extremely challenging.

To address the problem of stress separation in full-field photoelasticity measurements, several approaches have been proposed, all of which combine theoretical or numerical analysis and inverse approaches^1^,^2^,^3^,^4^. More recently the combination of digital photoelasticity combined with interferometric approaches has been experimentally realised which allows a complete experimental solution to the problem of stress separation^5^,^6^,^7^,^8^. For example, both Lei *et al*.^9^ and Yoneyama *et al*.^10^ have demonstrated stress separation in two-dimensions by combining circular polarimetry and interferometric photoelasticity. In their approach the phase values of the isochromatics and isoclinics are determined using a circular polariscope, whilst the phase of the isopachics is obtained using interferometry. The use of multiple interferometry measurements is required to account for phase shift errors caused by linear and quadratic reference phase deviations.

Ptychographic coherent diffractive imaging (CDI) is an iterative technique for quantitatively reconstructing the amplitude and phase of the complex wavefield exiting a sample^11^. In ptychographic CDI, instead of an in-focus image, the intensity distribution in the far-field associated with coherently scattered photons from the sample is recorded. Direct inversion of this diffraction pattern is impossible since the unique phase information accompanying the scattered wave is lost during its detection. As a result, an iterative phase retrieval process must be applied to recover the phase map and hence recover an image of the object under investigation. Unlike conventional visible light microscopy or other types of phase contrast optical microscopy such as Zernike, CDI recovers the quantitative amplitude and phase information of the wave exiting the sample (similar to Fourier Transform holography). Crucially for the present application, ptychography is able to simultaneously reconstruct the exit surface wave for both probe and object separately, entirely eliminating the need for multiple interferometry measurements^12^. In the X-ray regime ptychography has already been applied to a range of applications within materials science^13^,^14^. Recently Ferrand *et al*.^15^ showed in simulation the potential for anisotropic ptychography to recover birefringent sample information, however the combination of ptychography and optical photoelasticity has not been experimentally reported in the literature.

In this paper we demonstrate that a straightforward approach to quantitative in-plane stress determination can be achieved by combining the circular polariscope with ptychographic coherent diffractive imaging (CDI). Unlike previous approaches to stress separation, only one experimental setup is required for analysis of the phases associated with the isochromatics, isoclinics and isopachics. In addition to the ease of the optical set up, ptychography offers a number of other advantages when compared to previous interferometric based approaches:

(1) There are no errors due to non-linearity or misalignment of the set up leading to a higher quality result for the isopachic parameter.

(2) Ptychography can cope with complex, highly scattering (though not multiply scattering) samples which can cause problems for interferometry.

(3) For very small (sub-mm) specimens the total time to collect the data (including sample translations) can be less than for interferometric based set ups.

To confirm the validity of our proposed method, in common with Lei *et al*. and Yoneyama *et al*.^9^,^10^, we apply our method to the model problem of a diametrically compressed elastic disc. Unwrapping of the phases associated with the isochromatics and isopachics is readily achieved using a ‘quality guided’ phase unwrapping algorithm based on the approach of Ghiglia *et al*.^16^. The validity of our approach is verified by direct comparison of the experimental results to the analytic solution for a stressed disc under point loading first derived by Hertz^17^.

## Methods

### Experimental setup

**Figure 1:**
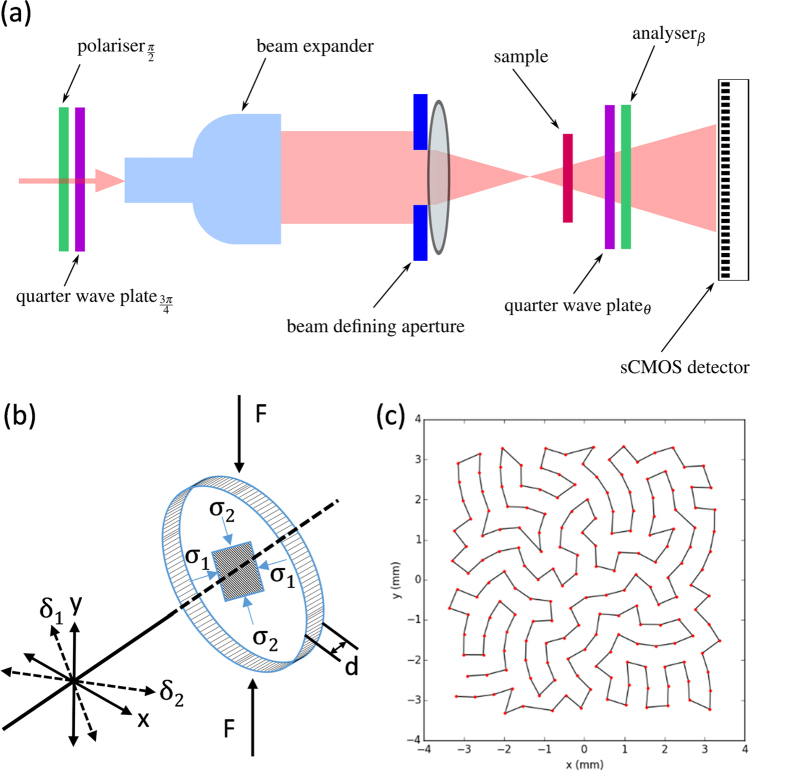
(**a**) Experimental set up for the ptychographic circular polariscope. (**b**) Stress-optic relationship for a birefringent stressed disc. (**c**) Fermat spiral pattern for ptychographic scan.

The combination of a circular polariscope and the imaging geometry described by Fresnel Coherent Diffractive Imaging (FCDI) Ptychography^18^,^19^,^20^,^21^ was used for the optical measurements (Fig. 1a). In this geometry, a laser beam (wavelength = 633 nm) passes through a polariser *P*_π/2_ whose polarised axis is set at a right angle of π/2, and a quarter waveplate *Q*_3π/4_ whose fast axis is set at an angle, 3π/4, with respect to a reference x-axis . The circularly polarised beam then enters a 20× beam expander which produces a 50 mm diameter parallel beam. A 15 mm diameter beam defining aperture (BDA) is then used to remove any parasitic scatter and select the central portion of the beam. The BDA was placed directly upstream of the focusing lens which consisted of a Thorlabs achromatic doublet with 50 mm diameter and 75 mm focal length. The sample, a 1 mm thick polycarbonate disc of 5 mm diameter was placed 30 mm downstream of the focus and was diametrically compressed using a force of 3.85 N in a custom built loading rig (Fig. 1b).

After exiting the birefringent sample *R*_δ,φ_, the beam passed through a second quarter waveplate *Q*_θ_ and an analyser *A*_β_. Both quarter waveplate and analyser were mounted on motorised Thorlabs rotation stages for selection of the polarised axis of the analyser and fast axis of the waveplate, which were set to arbitrary angles of *β* and *θ* respectively. The far-field diffraction patterns containing information on the isochromatic, isoclinic and isopachic fringes were then recorded on a water cooled Andor Zyla 4.2 sCMOS detector placed 190 mm downstream of the focus. The detectors sensor contains 2048 × 2048 pixels with a pixel pitch of 6.5 um. To improve signal-to-noise, four adjacent pixels were summed reducing the array size to 512 × 512 pixels. This optical geometry produces a 7.5 micron pixel size in the sample plane and a recovered field of view of 3.84 mm in each orthogonal direction (*x*, *y*). From this geometry, the beam size in the sample plane is approximately 1.56 mm in diameter accounting for an oversampling factor of 2.46.

### Data collection

For the ptychography scans multiple diffraction datasets are collected from overlapping measurement points on the sample. The overlap of adjacent measurement points means that the same sample features are present in multiple datasets. This has two positive benefits. Firstly it makes the reconstruction more robust and typically produces higher quality images than CDI. Secondly when combined with the extended Ptychographical Iterative Engine (ePIE)^22^ it enables the amplitude and phase of the illuminating probe to be reconstructed simultaneously with the sample. In the case of determining the in-plane tensorial strain components this latter feature offers significant benefits since the complex probe function can be thought of as being analogous to an unstrained reference measurement. In the case of phase stepping interferometry removing contributions to the phase due to linear offsets of imperfections in the set up can lead to spurious artefacts in the data^23^,^24^. Here the separation is handled by the ePIE algorithm^22^ allowing for an elegant solution to the problem of having a reliable reference.

The reconstruction of the phase information in ptychography is achieved by propagating the diffracted wavefield back-and-forth between the exit surface plane of the sample and the detector. Convergence of the algorithm is achieved by enforcing consistency with the measured intensity information at the detector and with information about the overlap of measurement points in the sample plane. Typically hundreds of iterations are required before the final image of the complex sample is obtained which on a modern high-performance computing workstation (as was used in the present case) takes between a few seconds to a few minutes (depending on the scan size). A detailed mathematical description of ptychography can be found in the following refs 11,12,25.

**Figure 2:**
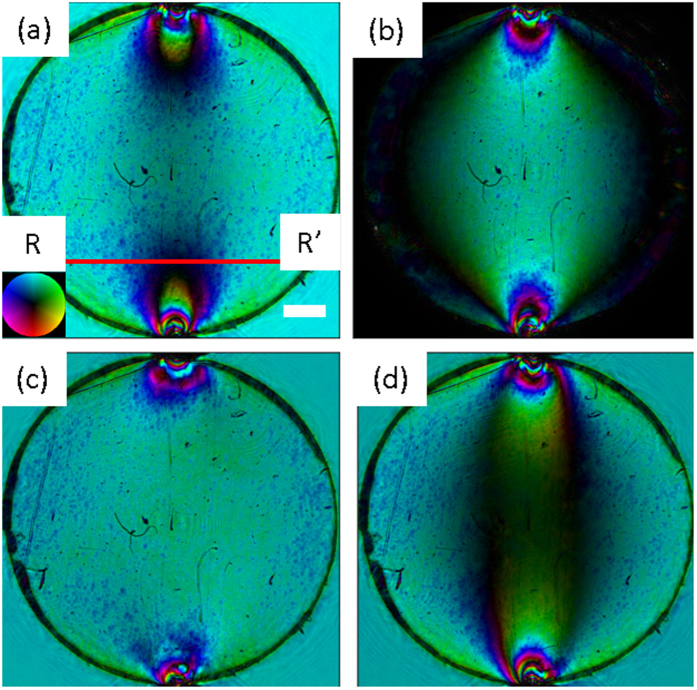
Four ptychographically reconstructed photoelastic light intensities corresponding to the optical configurations summerised in Table 1. The image brightness corresponds to the amplitude, whilst the hue corresponds to the phase information. Note the white scale bar in (**a**) is 0.5 mm.

**Figure 3:**
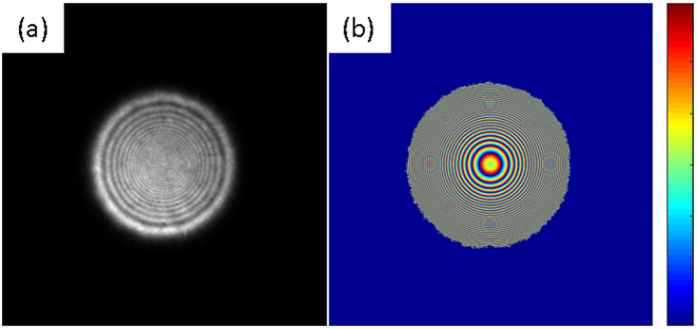
Complex probe reconstruction (**a**) intensity and (**b**) phase. Note the phase is only shown where the intensity is above 1% of its maximum value.

The ptychography data were collected in a Fermat spiral pattern in order to eliminate raster grid artefacts (Fig. 1c)^3^. The average distance between scan points was ~480 um, representing an overlap factor of ~70%. The total number of data points in the scan was 180 and the exposure time per point was 0.5 s giving a total exposure time of 90 s which equates to 11 min data collection time when motor moves are included. We note that although for the large samples presented here the ptychographic scan takes longer than the equivalent interferometry measurement, for smaller samples at higher resolution and with less scan points ptrychography could be faster. For example, to image an area of 30 × 30 μm currently takes 12 s using ptychography, this is generally faster than the 5 measurements at different phase offsets required for phase-stepping interferometry. For our ptychographic scans an optimisation was performed such that the scan points sequence minimised the total distance travelled. Image reconstructions were performed using the ePIE algorithm^12^. Due to the slowly varying sample phase, convergence of the ePIE algorithm was comparatively slow. However, as can be judged by the recovered phase information in Fig. 2 the quality of the sample reconstruction was good. The reconstructions were also aided by the presence of sharper features on the sample surface such as scratches and shallow dents created during sample fabrication. All reconstructions were completed within 500 iterations with the probe not being updated for the first 50 iterations, after which the probe was allowed to update at every iteration. An example reconstruction of the probe is shown in Fig. 3.

### Jones calculus for light intensity

Propagation of light through the various optical elements in Fig. 1 may be described using Jones calculus. The Jones matrix for a birefringent sample (*R*_δ,φ_) with phase retardations along the principal stress axis, denoted as *δ*_1_ and *δ*_2_, may be expressed as:
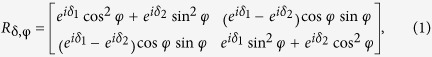
where *φ* is the angle that the maximum principal stress component makes with the reference axis. For the quarter waveplates whose fast axes make an angle *θ* and 3π/4 the Jones matrices are:
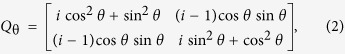

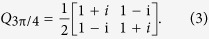


The Jones matrix for the analyser (a linear polariser) whose axis is set at an angle β is:
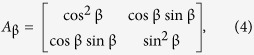


and in the case of the first polariser (*P*_π/2_) which produces vertically polarised light with respect to *x*:
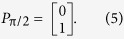


Finally to calculate the output wave vector, or exit surface wave, of the configuration, *a*′ we multiply the elements in order;
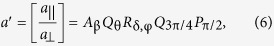
where the components (*a*_*||*_, *a*_⊥_) of the light vector *a*′ are directed parallel and perpendicular to the analyser axis respectively. The light intensity of the Jones vector *a*′ is then given by:
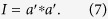
where *a*^′*^ represents the complex conjugate of *a*′. Using these Jones matrices we can calculate the intensities of the configurations for use in determining the phase of the isochromatics and isoclinics. The generalised intensity equation for the setup obtained from Eq. (7) is;
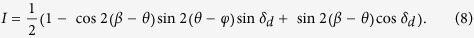
where the substitution *δ*_d_ = *δ*_2_ − *δ*_1_ has been made. Equation (8) was then used to generate formulae for the light intensities *I*_1_, *I*_2_, *I*_3_ and *I*_4_ corresponding to the four polarisation configurations as shown in Table 1.

### Phase analysis for isochromatics, isoclinics and isopachics

The four light intensities in Table 1 were used to determine the phase of the isoclinics (*φ*) and isochromatics (*δ*_d_) via:
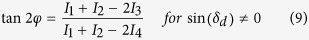


and
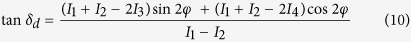


The determination of the phase of the isopachics (*δ*_*S*_ = *δ*_1_ + *δ*_2_) normally involves a separate interferometric based experiment consisting of multiple phase steps. However, ptychography allows one to simultaneously reconstruct probe and object function providing a quantitative phase for the isopachic parameter relative to the incident light. This fifth configuration used to measure the isopachics is obtained using *θ* = π/4 and *β* = π/2 placing the polariscope in so-called brightfield mode. Using Eq. 6 to calculate the complex output vector, 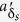, for the brightfield configuration produces the following matrix equation:
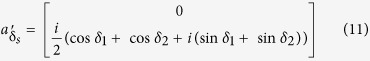


This matrix can be decomposed into its real and imaginary parts to give:
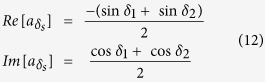


The amplitude and phase of the brightfield image are:
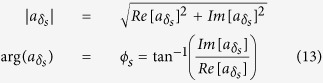


Substituting from Eq. (12) into Eq. (14) gives a value for the brightfield phase of:
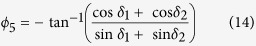


Which employing the simple trigonometric identity for cot and making the substitution for *δ*_*S*_ gives:
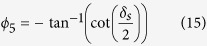
where *ϕ*_5_ is the ptychographically reconstructed phase in the brightfield configuration. Upon re-arrangement, we have that:
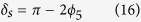
demonstrating that in the brightfield configuration, the ptychographically (or interferometrically) determined phase is directly related to *δ*_*S*_.

Unfortunately, the brightfield configuration is a special case where the measured phase is directly related to the isopachics. Determining the isochromatics and isoclincs requires a minimum of two additional polarisacope configurations are used. Since the measured phases depend on combinations of the isochromatic (*δ*_*d*_) and isoclinic (*φ*) phases, solving for these parameters requires trigonometric operations to be applied to the reconstructed sample images which in turn can lead to instabilities in the result.

Whilst the phase value of *φ* from Eq. (9) lies in the range [−*π*/4, *π*/4] the actual isoclinic parameter lies in the range [−*π*/2, *π*/2]^10^. Furthermore, the phase values of both the isochromatics and isopachics (Eqs 9 and 16 respectively) also require unwrapping. To unwrap the phases of the isoclinic, isochromatic and isopachic phase maps we used a 2D quality-guided phase unwrapping algorithm; in this approach the variance of the partial derivatives of the locally unwrapped phase is used to guide the phase unwrapping path^16^,^26^.

### Formulae for in-plane stress components

Using the unwrapped phase values of the isochromatics, isoclinics and isopachics the in-plane stress components can be calculated by relating the sum and difference of the principal stress components, *σ*_1_ and *σ*_2_, to the optical constants, A and B, wavelength of light, *λ*, and the thickness, *d* of the sample.
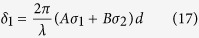

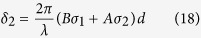


The optical constants for polycarbonate (Lexan) used in the present experiment have been previously tabulated as *C* = *A* + *B* = 34.6 × 10^−12^ Pa^−1^ and *D* = *A* − *B* = −165 × 10^−12^ Pa^−1 ^27. With the principal stress components calculated, the stress components in the lab reference frame can be determined:
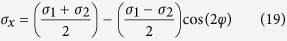

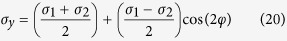

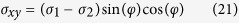


## Results and Discussion

To validate the experimental results from this study we compared our data to the analytic solution for the elastic stress distribution in a diametrically compressed disc first derived by Hertz in 1895^17^. This solution assumes negligible deformation of the edges of the disc which, through comparison with finite element modelling (FEM), we determined to be valid up to loads of at least 5.5 N, well above the experimental loads used here.

**Figure 4:**
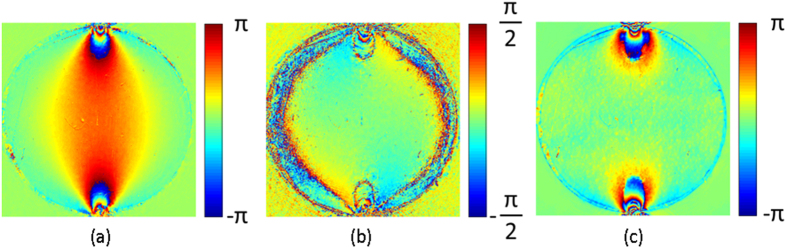
Wrapped phase maps of (**a**) isochromatics, (**b**) isoclinics and (**c**) isopachics.

Ptychographic reconstructions were carried out for all five optical polarisation configurations described in sections *2.3*–*2.4*. Figure 2(a–d) shows the complex reconstructed sample transmission functions corresponding to the first four optical configurations in Table 1. It is important to note that in this paper we have followed the approach described in refs 9, 10 based on the intensity information alone to calculate the isoclinc and isochromatic phases. As discussed in the Methods section in principle, since ptychography provides the phase in addition to the amplitude it should be possible to obtain the same information using just two additional measurements to determine both the isoclinc and isochromatic phases. In practice however, the relationship between *φ*, *δ*_d_ and the reconstructed phases, which can be derived using Jones calculus, involves more analysis steps than the equivalent formulae involving the intensity. This is in contrast to the relationship between *δ*_s_ and the reconstructed phase in the brightfield configuration. This means that extraction of the relevant isoclinic and isochromatic parameters necessitates several trigonometric operations as opposed to just one. The additional steps appear, at present, to result in a worse result for the isoclinc and isochromatic parameters, hence for *φ* and *δ*_d_ we used the intensity information alone^9^,^10^. The isoclinic and isochromatic phases calculated using Eqs 10 and 11 are shown in Fig. 4(a,b).

**Figure 5:**
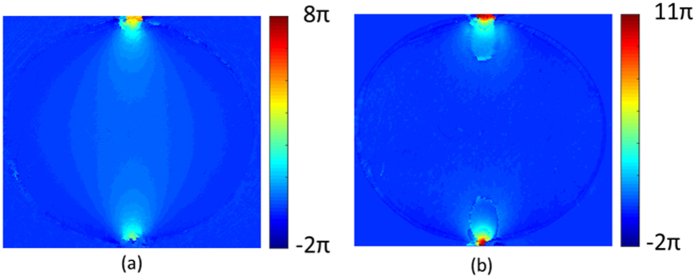
Unwrapped phase maps of (**a**) isochromatics and (**b**) isopachics.

Determination of the isopachic parameter can be carried out directly from the reconstructed phase in brightfield mode; this is a significant advance over previous phase stepping approaches which require a series of measurements (minimum of 5) to be performed on an interferometric set up. The ptychographically reconstructed isopachic phase is shown in Fig. 4(c). Unwrapping of the isoclinic, isochromatic and isopachic phases using the quality-guided algorithm resulted in smoothly varying phase maps across the sample (see Fig. 5), with the exception of close to the load points where the stress gradient is very large. These results are consistent with those reported in the literature^9^,^10^.

**Figure 6:**
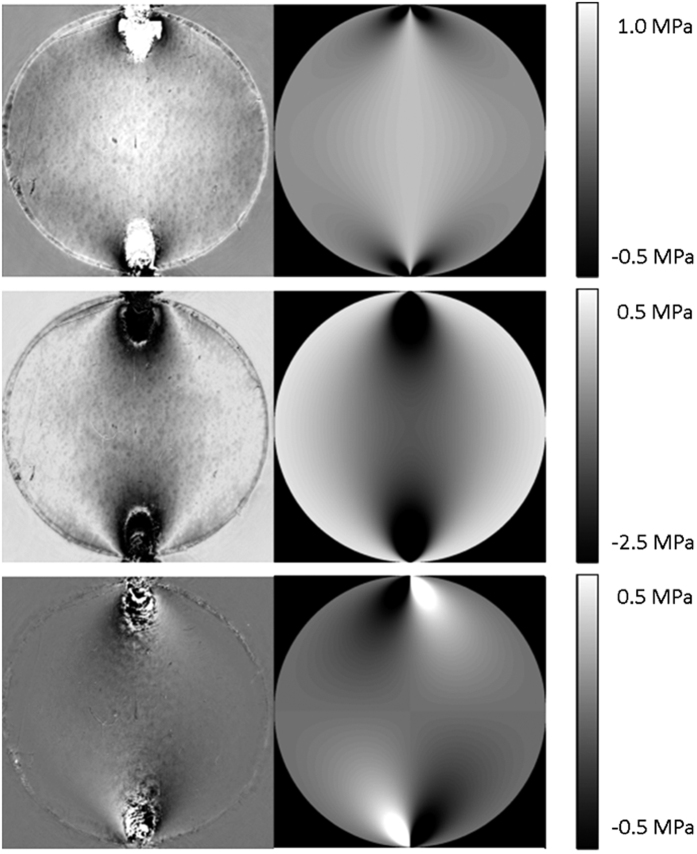
Reconstructed profiles for (a) experimental *σ*_x_ (b) theoretical *σ*_x_ (c) experimental *σ*_y_ (d) theoretical *σ*_y_ (e) experimental *σ*_xy_ and (f) theoretical *σ*_xy_.

**Figure 7:**
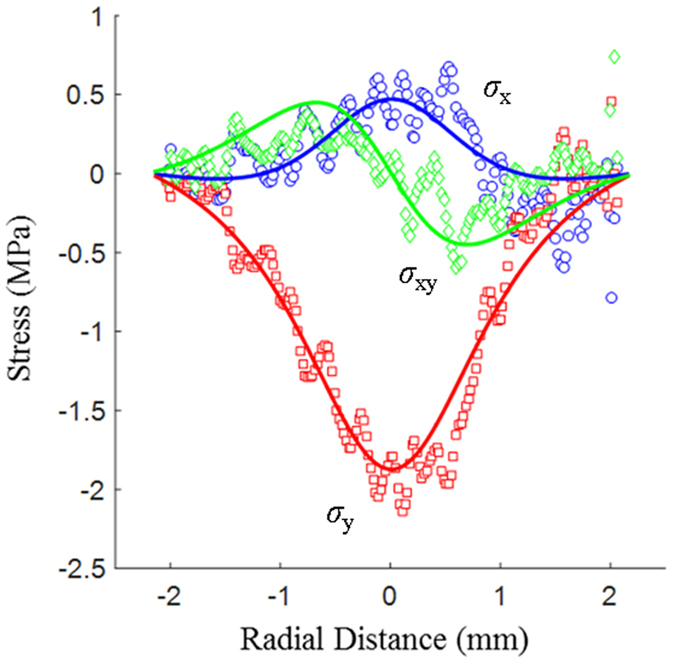
Plot of the experimental and theoretical stresses measured across the R–R’ line as depicted in Fig. 2(a). Solid lines are the theoretical curves obtained from the Hertz model and are overlaid with the experimental data for *σ*_x_ (blue circles), *σ*_y_ (red squares) and *σ*_xy_ (green diamonds).

Having obtained the unwrapped distributions for the isochromatics, isoclinics and isopachics, the experimental stress distributions quantified by *σ*_x_, *σ*_y_ and *σ*_xy_ were calculated. In Fig. 6 the results for the separate stress components obtained from ptychography are compared directly to the analytic solutions obtained from the Hertz model. Figure 7 compares the theoretical and experimental stresses calculated along the R–R′ (*y* = 1.25 mm) line. In this figure the solid lines represent the analytic solutions from the Hertz model calculated for a compressive load of 3.7 N. The load in the theoretical model was determined by iteratively updating the analytical Hertz solution until the best match was obtained to the experimental stress data. The match between the theoretical load and the experimentally applied one (3.85 N) is quite good. The ptychographically determined stress fields in Fig. 6 also appear to give a good match to the theoretically calculated values. We see that the largest difference between the two occurs close to the loading points. This greater mismatch in the immediate vicinity of the loading points is expected since the steep gradient in the stress fields make phase unwrapping more difficult within these regions. It is also clear that the edge of the polycarbonate disc contains some fabrication errors leading to slight perturbations from the ideal case. This is evidenced by the random oscillations in the experimentally determined strain values on the right hand side of Fig. 7. Taking these factors into consideration however, the data fit extremely well.

To quantify the quality of fit we have followed the approach of Krammer *et al*.^28^ and calculated the root mean square deviation (RMSD) normalised by the experimental data range – denoted NRMSD. Table 2 summarises the error analysis of five key parameters. When compared to the values of Krammer *et al*.^28^ four of these five values give a higher NRMSD. This is to be expected since the range of data in the case of Krammer *et al*.^28^ is approximately an order of magnitude greater than in the present case which, in general, will produce smaller relative errors. In addition, Krammer *et al*.^28^ use six separate intensity measurements to determine the values for (*σ*_1_ − *σ*_2_) and *φ* whereas in the present case we have used just four. The key result though is the one for the sum of the principal stresses which is proportional to *δ*_*S*_ and is therefore a direct indication of the quality of phase obtained using the bright-field ptychographic approach compared to the phase stepping approach. For this value, the NRMSD is actually improved compared to the previous work. This provides us with confidence that the direct phase determination using ptychography has the potential to deliver higher quality data than current phase stepping interferometric approaches.

The noise in the experimental data is typical of photoelasticity experiments for stress determination and is thought to arise from scratches on the disc surface and imperfections in the manufacture of the polycarbonate disc. Overall, the Cartesian experimental principal stress-fields have acceptable NRMSD errors ranging from 8.1% to 12.7%, demonstrating that the new ptychographic method is able to successfully determine the in-plane stress components for photoelastic materials.

## Conclusion

We have shown that photoelastic ptychography can be used to quantitatively measure the stress distribution within a diametrically compressed disc. The method used here is simpler than previous interferometric based techniques since all measurements are achieved using an in-line optical configuration. Reconstruction of the incident probe together with the complex sample wavefield allows for unambiguous retrieval of the isopachic parameter without the need for phase stepping. Combined with the previously established methods to calculate the isoclinics and isochromatics, the stress distribution throughout the sample is recovered. To the authors knowledge this represents the first experimental demonstration of photoelastic ptychography in the optical regime and, we believe, paves the way for diffractive imaging to be applied to a wide range of similar problems in the future.

## Additional Information

**How to cite this article**: Anthony, N. *et al*. A Direct Approach to In-Plane Stress Separation using Photoelastic Ptychography. *Sci. Rep.* **6**, 30541; doi: 10.1038/srep30541 (2016).

## Figures and Tables

**Table 1:** Light intensities for determining phase of isochromatics and isoclinics.

*θ*	*β*	*I*
0	π/4	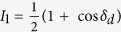
0	3π/4	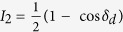
0	0	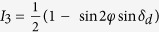
π/4	3π/4	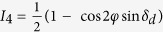

**Table 2:** Error analysis for key experimental parameters.

Quantity	Units	RMSD(in Units)	Data Range(in Units)	NRMSD(No Units)	NRMSD fromref. [28]
*φ*	rad.	0.22	1.98	0.111	0.075
*σ*_1_ + *σ*_2_	MPa	0.07	1.52	0.046	0.089
*σ*_1_ − *σ*_2_	MPa	0.35	3.20	0.109	0.026
*σ*_1_	MPa	0.16	1.26	0.127	0.075
*σ*_2_	MPa	0.19	2.34	0.081	0.066
